# Cohort and Trajectory Analysis in Multi-Agent Support Systems for Cancer Survivors

**DOI:** 10.1007/s10916-021-01770-3

**Published:** 2021-11-11

**Authors:** Gaetano Manzo, Davide Calvaresi, Oscar Jimenez-del-Toro, Jean-Paul Calbimonte, Michael Schumacher

**Affiliations:** University of Applied Sciences and Arts Western Switzerland (HES-SO), Institut Informatique de Gestion, HES-SO Valais-Wallis, Sierre, Switzerland

**Keywords:** Trajectory and cohort analysis, Machine learning, Multi-agent systems, Chatbots

## Abstract

In the past decades, the incidence rate of cancer has steadily risen. Although advances in early and accurate detection have increased cancer survival chances, these patients must cope with physical and psychological sequelae. The lack of personalized support and assistance after discharge may lead to a rapid diminution of their physical abilities, cognitive impairment, and reduced quality of life. This paper proposes a personalized support system for cancer survivors based on a cohort and trajectory analysis (CTA) module integrated within an agent-based personalized chatbot named EREBOTS. The CTA module relies on survival estimation models, machine learning, and deep learning techniques. It provides clinicians with supporting evidence for choosing a personalized treatment, while allowing patients to benefit from tailored suggestions adapted to their conditions and trajectories. The development of the CTA within the EREBOTS framework enables to effectively evaluate the significance of prognostic variables, detect patient’s high-risk markers, and support treatment decisions.

## Introduction

Breast cancer is the most common cancer in women worldwide and the second leading cause of cancer death for this population [1]. According to the American Cancer Society, the incidence rate of breast cancer has risen by $0.5\%$ per year, estimating 281, 550 new cases solely in the United States in 2021. From 2013 to 2018, medical advancements reduced the death rate by $1\%$ per year, increasing the cancer survival rate up to $90\%$ after five years from the diagnosis [2].

According to the World Health Organization (WHO), the improvement of treatment adherence would be more beneficial to the patient’s health than the development of new drugs [3]. However, the correct identification of some cancers’ stages and their evolution is still a challenging task [4], which often lead to insufficient or unnecessary treatments [5]. Therefore, the development of assistive technologies that *(i)* effectively evaluate the significance of prognostic variables (e.g., death or relapse), *(ii)* facilitate the detection of patient’s high-risk markers, *(iii)* support treatment decisions, and *(iv)* improve the patients’ treatment adherence, is imperative.

In this paper, we introduce a cohort and trajectory analysis (CTA) approach for the EREBOTS agent-based personalized chatbot system [6]. The CTA enables EREBOTS to support patients through treatment adjustment, and provides a dedicated interface for clinicians to fine-tune the chatbot behaviors. Numerical evaluations show the effectiveness of trajectory analysis for providing insightful prediction and classification results in the context of breast cancer survivor patient support.

The rest of the paper is organized as follows. “State of the Art” presents the state of the art followed by the open challenges. The multi-agents framework EREBOTS is introduced in “Architecture of the Agent-based Chatbot Platform”, together with its components, behaviors, and interfaces. The EREBOTS Cohort and Trajectory Analysis module is presented in “Model for Cohort and Trajectory Analysis”, whereas “Model Evaluations” provides its evaluation. Finally, “Conclusion” presents the discussions and conclusions of the paper.

## State of the art

Chatbot technologies are progressively being used to support cancer survivors [7]. Greer et al. [8] confirmed that technology can be an effective vector to reach young adults with positive psychology stimulation. As a result, the patients reported a sensible reduction of anxiety and depression. Chaix et al. [7] proposed Vik, a health care chatbot supporting breast cancer survivors. Although these works have shown the potential use of chatbots for supporting fragile cancer survivors, the challenge of personalizing interactions and interventions remains open. The usage of AI-powered models built upon patient information and their trajectories have been explored to fill this gap as seen in the next section.

Survival models evaluate the significance of prognostic variables in outcomes such as death or relapse, informing clinicians and patients of their treatment options [9]. The Kaplan–Meier estimator is one of the most used survival analysis models for cancer patients [10]. It allows establishing an estimation of the survival function from lifetime data, taking into account censored data and estimating lost event occurrence at a patient’s follow-up [11]. On the other hand, the Cox Proportional Hazard (CPH) model [12] is a standard method that can be adjusted with patient covariates using linear combinations [13]. In the past few years, researchers have developed non-linear models, based on deep learning architectures, to the problem of survival analysis [14]. In particular, they focused on neural networks (NN) for classification tasks [15], event estimations [16], and risk prediction [17]. Those neural networks learn highly complex and nonlinear relationships between prognostic features and individuals risks. However, previous studies have demonstrated mixed results on predicting risk, failing to demonstrate improvements beyond the linear Cox model [18, 19].

The studies previously presented intersect several disciplines and domains including patient trajectory analysis, conversational agents, and eHealth patient support. The opportunities arising from the combined synergy of these areas are: *(i)* the dissemination of health information and coaching instructions; *(ii)* the collection of patient data to enable profiling, monitoring, and adherence boosting interactions; *(iii)* the incentive of positive behavioral change; *(iv)* the support of persuasive strategies for self-efficacy evaluation.

Cancer survivors could concretely benefit from the accomplishment of such combinations. Equipping chatbots with behaviors bridging patients’ trajectories and persuasive techniques can support eHealth systems, which are facing the strain of a significant demand for patient empowerment.

## Architecture of the agent-based chatbot platform

To address the challenges mentioned above, we rely on an agent-based chatbot platform named EREBOTS. The multi-agent-based architecture of the platform allows autonomous execution of personalized behaviors towards patients and isolated management of personal data.

**Fig. 1:**
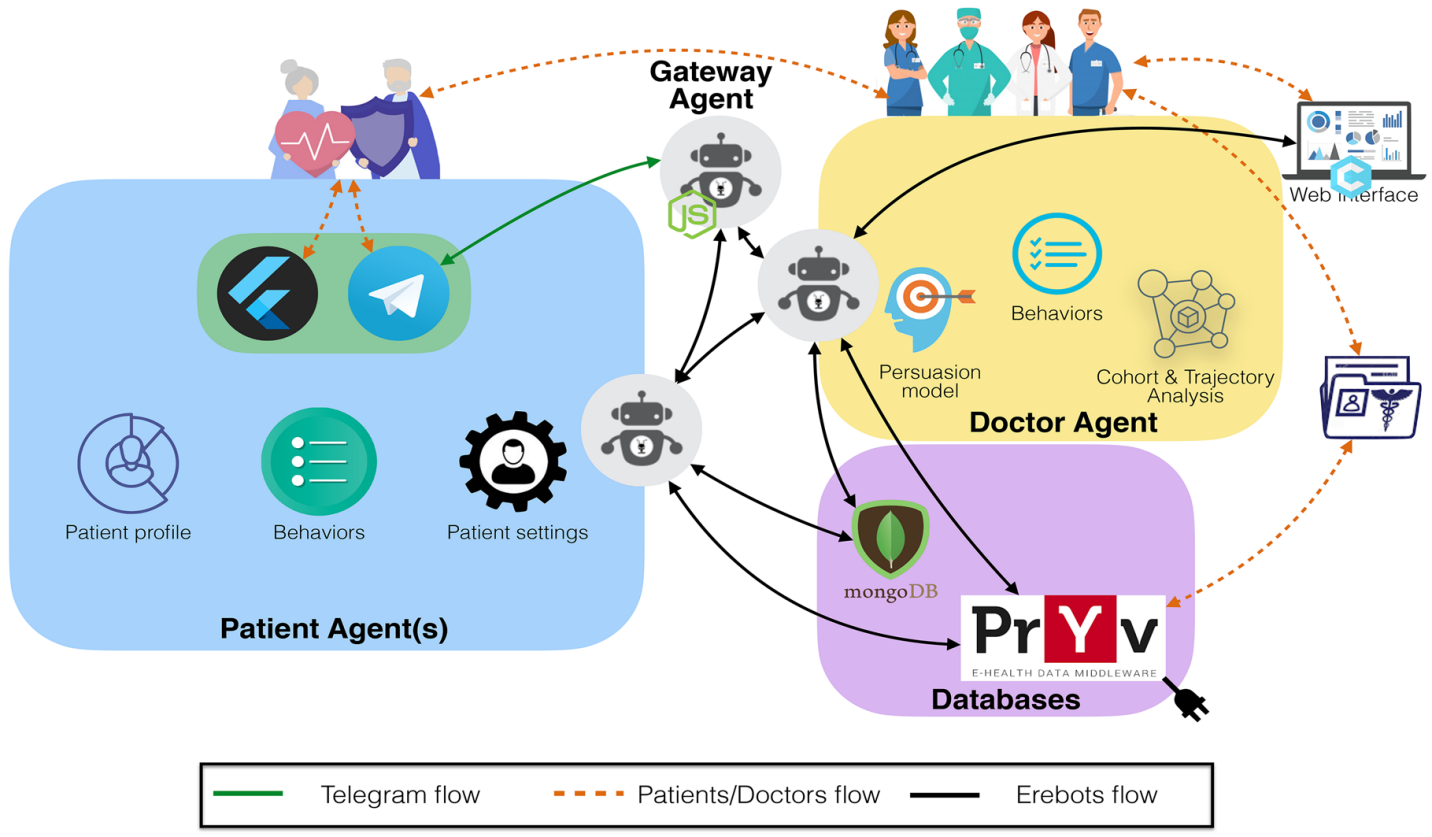
EREBOTS architecture and interactions containerized via Docker

Figure 1 shows the EREBOTS platform [6] comprises four main components: Database management, Communication Server, Multi-Agent System back-end for the doctor agents, and Multi-Agent System back-end and front-end for the patient agents.

- The *Database* component manages two types of information: *(i)* system-related (non-personal) data, managed through *MongoDB*, and *(ii)* user personal data through *Pryv*^1^.
- The *Communication server* for inter-agent communication within the Multi-Agent Systems (MAS) uses Prosody^2^, an XMPP server instance.
- The *Doctor agent* is designed to autonomously manage a campaign, including the type of interactions defined for the patients and the monitoring of their activities. It organizes the patients’ data, elaborates patients trajectories based on machine learning models, updates the forecast trends, and enables further analysis. Overall, the Doctor agent is characterized by three building-blocks: *Persuasion models* to foster the Patient(s) behavioral change, the agent set of *Behaviors*, and the *CTA* module.
- The *Patient agent* manages the patients’ connections and their messages from the chat platform(s).

Please refer to [6] for more details of the EREBOTS architecture and development.

## Model for cohort and trajectory analysis

In our approach, agents deployed in EREBOTS take either the patient or the doctor role, autonomously managing the interactions produced and received through the chatbot messages. Within the doctor agent, we propose the inclusion of the Cohort and Trajectory Analysis model, whose purpose is to provide decision-support information for clinicians regarding risks, symptoms, and disease associations.

**Fig. 2:**
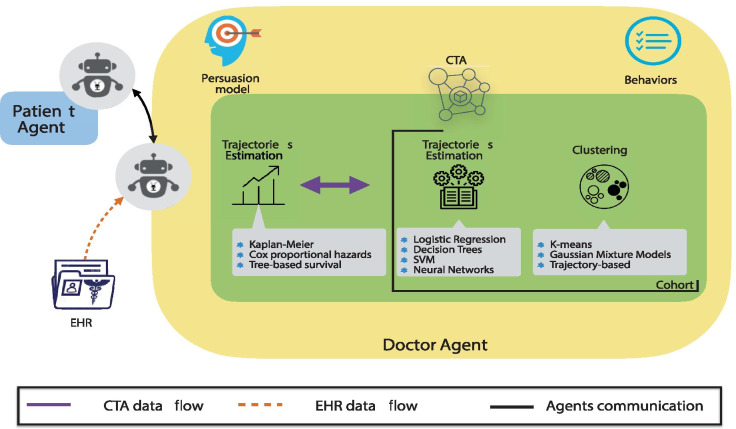
Cohort and Trajectory Analysis (CTA) architecture in EREBOTS

Figure 2 illustrates the general scheme of the trajectory and cohort analysis process. Specifically, trajectories represent the patient’s evolution from the diagnosis of the disease. The CTA requires EHRs data, provided by the doctor-agent, and behavioral data, provided by the patient-agent. Through the analysis of the trajectories, it is possible to identify associations between symptoms and events, and to quantify risks. Finally, these results allow the identification of high-risk markers for detrimental treatment effects, subsequent cancer disease, and metastatic cancer disease.

### Trajectory estimation

In the context of cancer survivorship, one key aspect is the prediction of life expectancy, related to the probability of cancer relapse. To address this challenge, we introduce survival models, which aim to answer the question: “what is the probability that a patient survived any time *t*?.” We denote the survival function as $S(t)=Pr(T>t)$ where *T* is the time of an event, and *t* is the time from the beginning of an observation period to an event. Please notice that $S(t)=1$ when $t=0$, whereas $S(t)=0$ when $t=\infty$. In case the study ends or the patient is withdrawn from it, the data is considered *censored*. Given a dataset with patients observing time and event outcome, we are enabled to estimate the survival curve through the Kaplan-Meier Estimator [11]:

1 $$\begin{aligned} S(t)= \prod _{i=0}^t 1-Pr(T=i,t>=i)=\prod _{i=0}^t 1-\frac{d_i}{n_i}. \end{aligned}$$

With $d_i$ and $n_i$ the number of patients that had an event at time *i* and the number of patients that survived at time *i*, respectively. Please note that the Kaplan-Meier estimator formula is obtained by using the chain rule for random variables. Indeed, the Kaplan-Meier estimator is calculated considering the notion that the probability can be broken up into the product of probabilities during specific intervals.

To provide personalized patient treatments, we need to evaluate the hazard function that analyzes individual risks answering the question: “What is the immediate death risk for a patient that survived at time *t*?”.

The Cox Proportional Hazard model provides the tool to estimate individual risks as follows:

2 $$\begin{aligned} \lambda (t)=\lambda _0e^{(factor)} \end{aligned}$$

where *t* is the observation period and $\lambda _0$ is the baseline risk. Whereas, the *factor* in 2 identifies the way of modeling patient features (e.g., age, tumor stage, and treatments) to estimate patient risk. In this work, given its performance, we define the factor risk as a linear combination of the patient’s features $X=(x_1,x_2,..,x_n)$ and the respective features’ weights $\Theta =(\theta _1,\theta _2,..,\theta _n)$, with *n* the number of patient features.Therefore,

3 $$\begin{aligned} \lambda (t)=\lambda _0e^{(\theta _1x_1,\theta _2x_2,..\theta _nx_n)}=\lambda _0e^{\left( \sum _{i=0}^n\theta _ix_i\right) }=\lambda _0e^{( \Theta ^tX)}. \end{aligned}$$

Please notice that the survival function 1 is strictly related to the hazard function, as follows:

4 $$\begin{aligned} S(t)=e^{-\int _0^t\lambda (u)du},\end{aligned}$$

and vice versa

5 $$\begin{aligned} \lambda (t)=-\frac{S'(t)}{S(t)}\end{aligned}$$

### Survival classification

The survival and risk approaches described above provide the means for doctor agents to build a comprehensive trajectory model that can be used to support clinical decisions. Complementary to these features, we propose incorporating classification prediction capabilities, which may help understanding an individual trajectory based on similar healthcare records.

To identify common patterns of patient data, we define a classification task based on the use of several machine learning and deep learning models. These survival classifiers identify the relationship of the features based on the patient outcome event (e.g., death), while providing interpretable results. They take as input the data of the patients (e.g., cancer type, tumor stage, and Nottingham Prognostic Index) and provide as output the label group to which the patient belongs, such as the patient’s vital status or relapse-free status.

Notice that this first step to cluster patients with similar features requires a training phase (i.e., supervised learning), contrary to clustering approaches presented later (i.e., unsupervised learning).

The classification itself starts first with the task of finding the event probability of the dependent binary variable (outcome), e.g., to be alive or deceased. To understand the decision boundary of the classification task, we use Support Vector Machine. Decision trees, random forests, and stochastic gradient boosting are the tree-based models used for the classification as well for the trajectory analysis tasks. Those models handle continuous and categorical features, outliers, and missing data. Finally, as the last classifier, we use a deep learning model —Neural Networks, which show high accuracy for large datasets.

### Clustering

While in the previous section the classification methods targeted prediction on predefined trajectory outcomes, in this section we investigate different clustering approaches for the patient cohort analysis. Such unsupervised approaches benefit from the trajectory analysis and overcome the limitations of the classification task, which is based on the outcome of events such as the vital or the relapse status of the patient. Using clustering, a doctor agent can find similar trajectories without pre-defined assumptions about their patient’s characteristics.

To fine-tune groups of patients with similar outcomes but different features, we use K-Means, Gaussian Mixture Models, and Trajectory-based clustering algorithms.

## Model evaluations

To evaluate the Cohort and Trajectory Analysis module, we have applied it in the context of breast cancer support. The fundamental principle is that these models can autonomously provide personalized prediction of risks, as well as classification of trajectory patterns to incorporate into an EREBOTS doctor agent. In the following, we describe first the dataset used during the evaluation followed by the trajectory and cohort analysis results.

### Dataset

In order to train and evaluate our models, we use the METABRIC dataset (Molecular Taxonomy of Breast Cancer International Consortium [20]). The METABRIC dataset consists of gene expression data and clinical features for 2, 498 patients labeled as follows: $33.34\%$ *“Living”*, $25.74\%$ *“Died due to breast cancer”*, $19.80\%$ *“Died due to other causes”*, and the rest *“not observed”*. METABRIC includes 32 features such as age at diagnosis, type of breast surgery, and ER status. Moreover, the dataset presents the number of patient’s months of relapse-free status and overall survival status, respectively with a median relapse time of 99 months and survival time of 116 months. The mean age at diagnosis is 60, with the youngest patient at the age of 21 and the oldest at the age of 96.

### Trajectory analysis

**Fig. 3:**
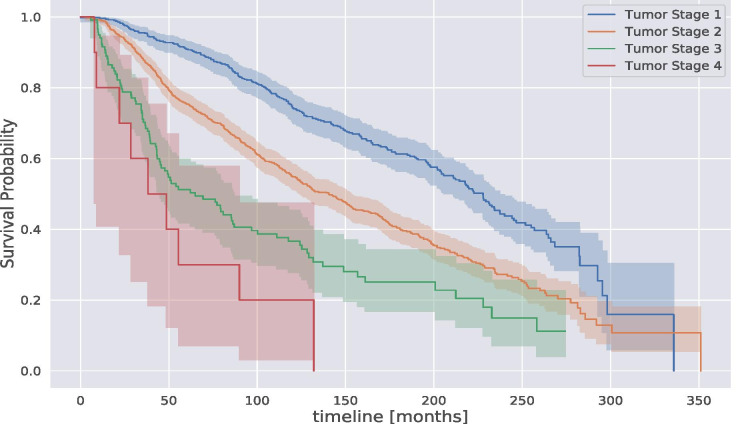
Kaplan-Meier survival probability estimation of the breast cancer population in METABRIC grouped by tumor stage

In this section, we illustrate the main results of our trajectory analysis. We start analyzing the METABRIC dataset using the Kaplan-Meier estimator (KM). Figure 3 shows the KM estimation of the breast cancer population grouped by tumor stage. We can see the impact of the tumor stage on the survival probability. Indeed, while a stage 2 tumor patient has a similar trend to the overall KM estimation above presented, patients with a stage 4 tumor have a huge drop in the survival probability estimation. According to our estimation, $80\%$ of patients with a tumor stage 2 survived at least 50 months, whereas only $40\%$ of patients with tumor stage 4 survived at least 50 months and just a few percentages survived more than 10 years.

**Fig. 4:**
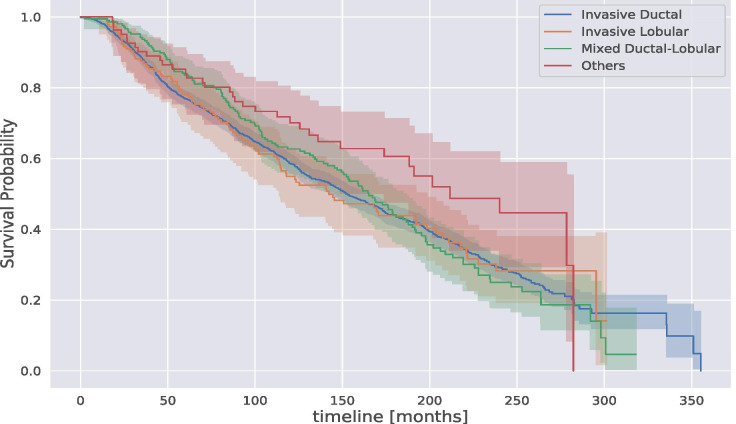
Kaplan-Meier survival probability estimation of the breast cancer population in METABRIC grouped by cancer type

In Fig. 4, we report the KM estimation of the breast cancer population grouped by cancer type. In particular, we arranged 4 types of cancers: Invasive Ductal, Invasive Lobular, Mixed Ductal-Lobular, and other types that represent less than $5\%$ of our dataset. From our KM estimation, we notice that all types of breast cancer in our dataset report the same KM estimation except for the group “Others”, which reports the best survival probably. However, given the small population and the numerous missing data, the KM estimation for the group “Others” presents a huge confident interval and a survival probability drop at 250 months.

**Fig. 5:**
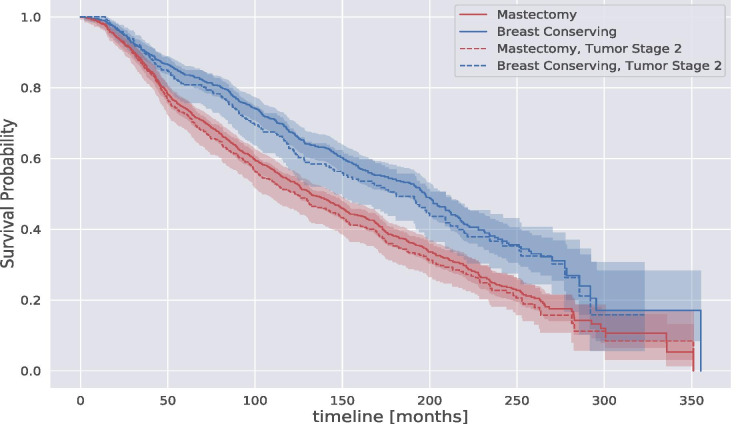
Kaplan-Meier survival probability estimation of the breast cancer population in METABRIC grouped by surgery type and tumor stage

A significant difference in the survival probability concerns the type of surgery. Figure 5 shows the KM estimation for the breast cancer population grouped by two surgeries: mastectomy and breast-conserving. We can see that breast-conserving surgeries provide a higher survival probability than mastectomy surgeries even for patients with similar tumor stages. Indeed, in Fig. 5, we report the KM estimation of both surgeries for patients with stage 2 tumors. We notice that tumor stages do not affect the survival trajectory estimation based on the type of surgery.

**Fig. 6:**
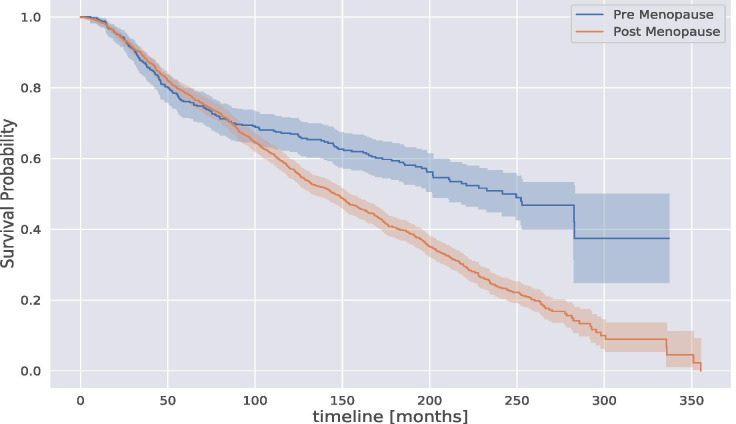
Kaplan-Meier survival probability estimation of the breast cancer population in METABRIC grouped by menopause status

A significant discrepancy in the survival estimation probability concerns the menopause status of the patients, which is strongly related to the patient’s age. Figure 6 shows the KM survival estimation of the breast cancer patients grouped by menopause status: pre-menopause and post-menopause. As Fig. 6 shows, patients post-menopause present higher risks than patients pre-menopause due particularly to the elder breast cancer population in the post-menopause group.

**Fig. 7:**
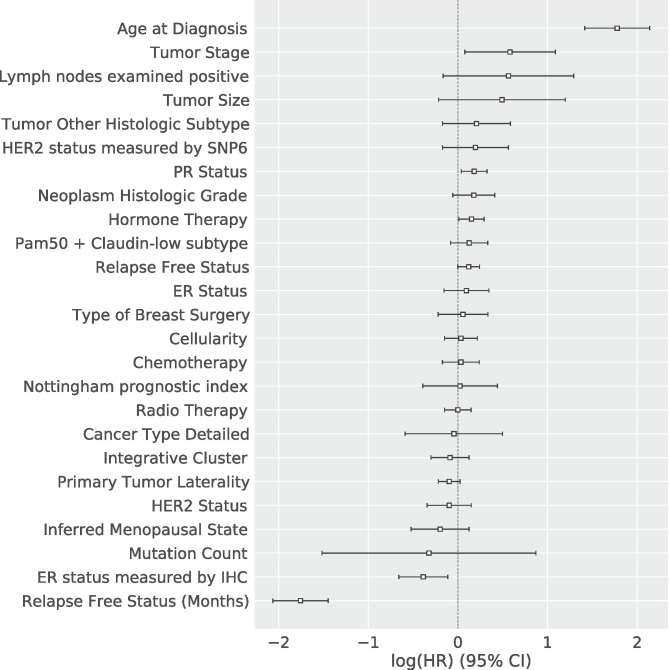
Cox Proportional Hazard based on the survival analysis of the breast cancer population in METABRIC

As a support to the relationship between patient’s age at diagnosis and patient’s risk, in Fig. 7, we present the Cox Proportional Hazard based on the survival analysis of the breast cancer population in METABRIC. Figure 7 illustrates the relationship of the features with the *log* of the hazard function presented in “Model for Cohort and Trajectory Analysis” and the respective confident interval of $95\%$. Please note that the defined hazard function is exponential (see Eq. 3), therefore, the relationship between the features and the *log* of the hazard function is linear. In such a plot, a positive relationship means higher risk. On the other hand, a negative relationship means lower risk. As shown in Fig. 7, the patient’s age at the diagnosis is strongly related to higher risk. On the other hand, Relapse Free Status is strongly related to lower risk.

### Cohort analysis

In this section, we illustrate the main results of our cohort analysis using the METABRIC dataset. As mentioned, we use supervised and unsupervised machine learning algorithms such as Logistic Regression, SVM, Decision Tree, and Neural Networks.

**Fig. 8:**
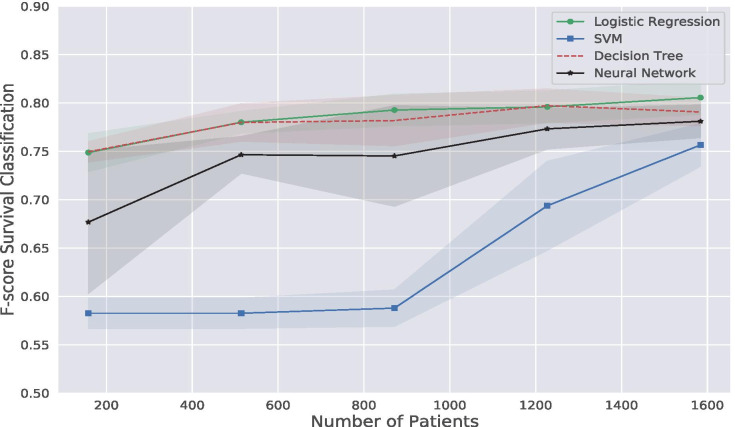
F-score for survival classification using Logistic Regression, SVM, Decision Tree, and Neural Networks

We start analyzing the learning curves of the different classifiers shown in Fig. 8. The figure illustrates the accuracy metric F-score for the survival classification task, defining patients’ risk, over the number of patients used for the training phase. We see that the classifiers enhance their accuracy when the number of patients in the training set increases. Particularly for the Neural Network model, which requires many examples to train its neurons. However, models such as Decision Tree and Logistic Regression provide the best accuracy level even for a small number of train examples.

Please notice that another important characteristic is the time to perform the cohort analysis. Indeed, while the trajectory analysis does not require any training phase, the cohort could imply some delay in the EREBOTS framework.

**Fig. 9:**
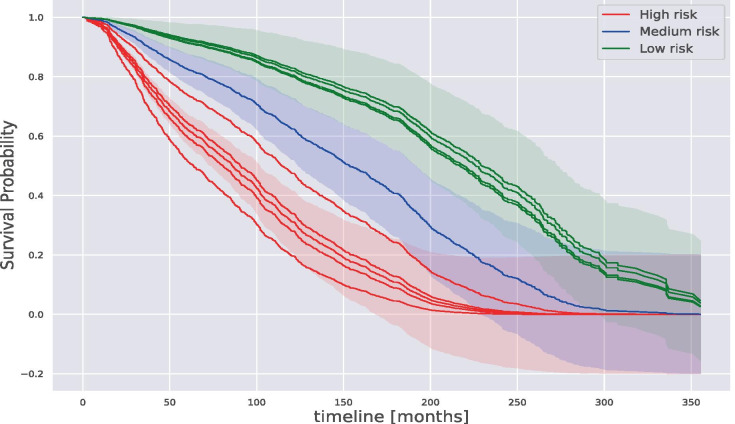
Gaussian mixture model patients cohort based on their trajectory

In Fig. 9, we show the Gaussian Mixture model performing the cohort analysis based on 10 random patient trajectories. We selected three main areas: high-risk, medium-risk, and low-risk. On the one hand, patients in the low-risk area have shown high survival probability. On the other hand, patients in the high-risk area have shown low survival probability. The Gaussian Mixture model clusters the patient trajectory in one of the above-mentioned areas defining the patient risk. The trajectory, based on the Cox Proportional Hazard model, takes into account the relationship among the patient covariates and the relationship between patients.

## Conclusion

This work coped with the challenge of personalized agent-based chatbots as virtual assistants for breast cancer survivals and clinicians’ support. In this context, we presented a Cohort and Trajectory Analysis (CTA) approach for survival estimation, risk prediction and classification, designed to be integrated as a module of the EREBOTS multi-agent chatbot framework.

Overall, the CTA enables EREBOTS to personalize mainstream interaction story-lines for dynamic personalization. By monitoring and reporting high-risk markers, the CTA provides support for the medical personnel for continuous healthcare supervision and prognosis. By using the CTA, our agent-based chatbots can model the users comprehensively for evolving patient models and behavior. The CTA can trigger an adjustment of the patient’s treatments for dynamic persuasive techniques.

As future work, we are investigating how to dynamically integrate user groups dedicated to enriching the chatbot interface (HemerApp) and its interactions. We wish to include in the CTA the mechanism presented in [21] and [22] for model ranking and accurate prediction on the overall survival of breast cancer patients.

## Footnotes

1 https://www.pryv.com/

2 https://prosody.im/
